# Insulin Signaling Pathway Mediates FoxO–Pepck Axis Regulation of Glucose Homeostasis in *Drosophila suzukii*

**DOI:** 10.3390/ijms251910441

**Published:** 2024-09-27

**Authors:** Shuting Zang, Ruijuan Wang, Yan Liu, Shan Zhao, Long Su, Xiaoyan Dai, Hao Chen, Zhenjuan Yin, Li Zheng, Qingxin Liu, Yifan Zhai

**Affiliations:** 1 Institute of Plant Protection, Shandong Academy of Agricultural Sciences, Jinan 250100, China; 2 Key Laboratory of Natural Enemies Insects, Ministry of Agriculture and Rural Affairs, Jinan 250100, China; 3 College of Life Sciences, Shandong Agricultural University, Tai’an 271000, China

**Keywords:** *Drosophila suzukii*, insulin signaling pathway, glycolysis/gluconeogenesis, FoxO, Pepck

## Abstract

The agricultural pest *Drosophila suzukii* exhibits a strong preference for feeding on fresh fruits, demonstrating high adaptability to sugary environments. Meanwhile, high sugar levels stimulate insulin secretion, thereby regulating the steady state of sugar metabolism. Understanding the mechanisms related to sugar metabolism in *D. suzukii* is crucial due to its adaptation to these specific environmental conditions. The insulin signaling pathway is an evolutionarily conserved phosphorylation cascade with significant roles in development and metabolism. We observed that the activation of the insulin signaling pathway inhibited FoxO activity and downregulated the expression of *Pepck*, thereby activating glycolysis and reducing glucose levels. By contrast, inhibiting insulin signaling increased the FoxO activity and upregulated the expression of *Pepck*, which activated gluconeogenesis and led to increased glucose levels. Our findings demonstrated the crucial role of the insulin signaling pathway in mediating glucose metabolism through the FoxO–Pepck axis, which supports the ecological adaptation of *D. suzukii* to high-sugar niches, thereby providing insights into its metabolic control and suggesting potential strategies for pest management. Elucidating these molecular processes is important for understanding metabolic regulation and ecological specialization in *D. suzukii*.

## 1. Introduction

*Drosophila suzukii* is a dipteran insect that belongs to the family Drosophilidae. *D. suzukii* originated in South East Asia, and it is an invasive, destructive crop pest [1]. Unlike *Drosophila melanogaster*, which prefers decayed fruit for oviposition, *D. suzukii* females utilize their saw-like ovipositors to penetrate ripening fruit for egg laying [2,3]. The developing *D. suzukii* larvae feed on the interior of the infested fruit and make it unsuitable for the market [4,5], thereby resulting in both direct and indirect economic impacts, including yield losses, infested fruit with a shorter shelf life, and increased production costs [6,7]. Ripe fruit, while abundant in sugars, lacks proteins and amino acids compared to decaying fruit, providing a nutrient-poor habitat for insects [4,8,9]. Therefore, elucidating the molecular mechanisms that maintain sugar metabolism homeostasis in *D. suzukii* is essential for developing pest biological control approaches.

Throughout insect growth and development, glucose homeostasis is controlled by various factors to ensure sufficient energy supply for development and to sustain general health [10]. When energy is required, glucose is metabolized to pyruvate through a series of enzymatic steps. The key enzymes involved in glycolysis include hexokinase (Hk) and pyruvate kinase (Pk), which catalyze the phosphorylation of glucose to glucose-6-phosphate and the conversion of phosphoenolpyruvate to pyruvate, respectively [11,12,13]. Pyruvate generated from glycolysis can further fuel cellular respiration or serve as a precursor for various biosynthetic pathways [14,15]. Prolonged fasting or starvation induces the production of glucose from non-carbohydrate precursors in the process called gluconeogenesis [16]. In this pathway, pyruvate carboxylase and phosphoenolpyruvate carboxykinase (Pepck) play pivotal roles in converting pyruvate to phosphoenolpyruvate [17,18], while glucose-6-phosphatase (G6p) catalyzes the conversion of glucose-6-phosphate to glucose [19]. Gluconeogenesis allows insects to maintain glucose homeostasis under conditions of fasting or low carbohydrate availability to ensure the availability of a steady supply of energy for vital physiological processes [20,21]. Furthermore, glucose metabolism in insects includes the regulation of glycogen metabolism [22,23], where glucose is stored in the form of glycogen to meet future energy needs. Glycogen synthesis is catalyzed by glycogen synthase (Gs), and glycogen phosphorylase (Gp) mediates the breakdown of glycogen [23,24] to release glucose when energy demands increase [25]. This dynamic regulation of glycogen metabolism allows insects to adapt to fluctuating energy requirements during different developmental stages or under environmental challenges [26,27]. In addition, insects possess specialized pathways for the synthesis and utilization of trehalose, which is a disaccharide comprising two glucose molecules [28,29,30]. In insects, trehalose functions as a critical energy source and stress protector [31], synthesized by trehalose-6-phosphate synthase (Tps) and degraded by trehalase (Tre) [32,33]. The ability to modulate trehalose levels in response to environmental cues is crucial for the survival of insects [34] and their adaptation to various stressors [35], including temperature fluctuations and desiccation [36]. Insects exhibit diverse glucose metabolism mechanisms to adapt to various ecological niches [37,38,39]. In particular, the insulin signaling pathway plays pivotal roles in the regulation of metabolic homeostasis and energy balance [40,41,42]. In both vertebrates and invertebrates, the insulin signaling pathway remains highly conserved [43]. The insulin (or insulin-like peptides (ILPs)) binds to its receptor on the target cell membrane; the receptor undergoes autophosphorylation, activating its intrinsic tyrosine kinase activity. This activation triggers a cascade of downstream signaling molecules, including insulin receptor substrate (IRS) proteins. Phosphorylated IRS proteins subsequently activate the PI3K (phosphoinositide 3-kinase)/Akt signaling pathway [44]. In *Maruca vitrata*, the hemolymph trehalose levels increase after the knockdown of insulin-receptor genes [45]. In *Aedes aegypti*, protein kinase B (Akt) depletion affects glucose metabolism by influencing the phosphorylation of 4E-binding protein, thereby leading to a reduced lifespan in adult females [46,47,48].

FoxO (Forkhead box O) is a key transcription factor involved in regulating various cellular processes such as metabolism, stress resistance, and longevity. In mammals, the FoxO family consists of FoxO1, FoxO3, FoxO4 and FoxO6 proteins [49]. In *D. suzukii*, there is a single FoxO homolog, whose function closely resembles that of its mammalian counterparts [50,51,52]. The evolutionary conservation of FoxO highlights its significance across species. The activity of FoxO transcription factors is tightly regulated by various signaling pathways, especially the IIS pathway [53]. In this pathway, the transcription factor FoxO is negatively regulated as a downstream target [54,55]. FoxO loses its transcriptional activity as it is translocated from the nucleus to the cytoplasm following phosphorylation by Akt, which is activated in response to insulin [56]. This dynamic regulation of the activity of FoxO is pivotal for coordinating cellular responses to changes in nutrient and energy conditions [57]. In mice, FoxO1 promotes lipid breakdown by regulating lipolytic genes such as *ATGL* and *HSL* in adipocytes, while also modulating glucose metabolism in peripheral tissues and pancreatic β cells [58,59]. Similarly, in *D. melanogaster*, FoxO inhibits lipid storage and promotes lipid mobilization by downregulating genes involved in lipid synthesis [54]. By influencing the expression of genes involved in gluconeogenesis, glycolysis, and insulin signaling, FoxO orchestrates a finely tuned regulatory network that governs glucose utilization and production [60,61].

In the present study, to explore the unique metabolic adaptations of *D. suzukii*, we focused on the pivotal role of the insulin signaling pathway in regulating glucose homeostasis via the FoxO–Pepck axis. We investigated the dual regulatory mechanisms associated with insulin signaling, which inhibited the FoxO activity and downregulated *Pepck* to promote glycolysis when the insulin signaling pathway was activated, whereas the inhibition of insulin signaling enhanced the FoxO activity and upregulated *Pepck* to stimulate gluconeogenesis. Our research indicates that insulin plays a crucial role in regulating glucose metabolism homeostasis in *D. suzukii*. This pathway provides a theoretical foundation for supporting the unique ecological adaptability of *D. suzukii*.

## 2. Results

### 2.1. Impact of High Sugar on Survival, Fecundity, and Insulin Content in D. suzukii

**Figure 1:**
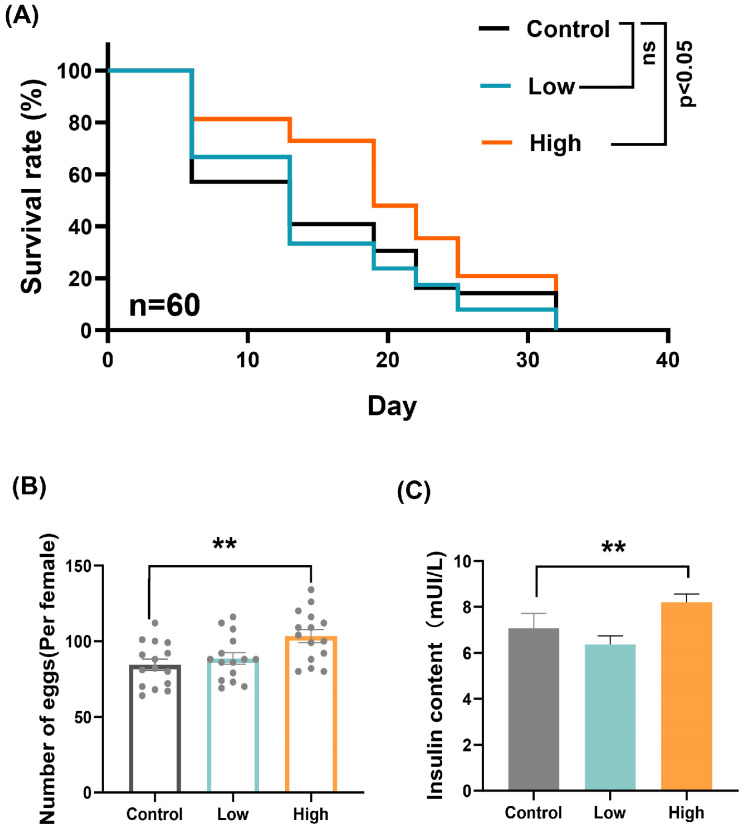
Impact of high sugar on survival, fecundity, and insulin content in *D. suzukii*. (**A**) Survival rates of *D. suzukii* adults under different sugar concentrations: control, low sugar and high sugar. (**B**) Fecundity of *D. suzukii* females under different sugar concentrations: control, low sugar, and high sugar. (**C**) Insulin content in *D. suzukii* under different sugar concentrations. Data represent means ± SE, with statistical significance indicated (ns: no significant, ** *p* < 0.01).

To explore the effects of different sugar levels on the growth of *D. suzukii*, we established two experimental groups: a low-sugar group and a high-sugar group. Firstly, we assessed the survival rates under each treatment. The results showed no significant difference in survival rates between the control group and the low-sugar group. However, the high-sugar group exhibited a significantly higher survival rate compared to the control group (Figure 1A). In addition, we measured the fecundity and insulin content in each group. The number of eggs per female was significantly higher in the high-sugar group compared to the control group (Figure 1B), indicating that a high-sugar diet enhances the reproductive capacity of *D. suzukii*. Furthermore, insulin content was significantly elevated in the high-sugar group compared to the control group (Figure 1C). These results indicate that *D. suzukii* shows better adaptability in high-sugar environments, with high sugar promoting insulin secretion.

### 2.2. Insulin Affects Carbohydrates and Expression of Genes Related to Glycometabolism in D. suzukii

**Figure 2:**
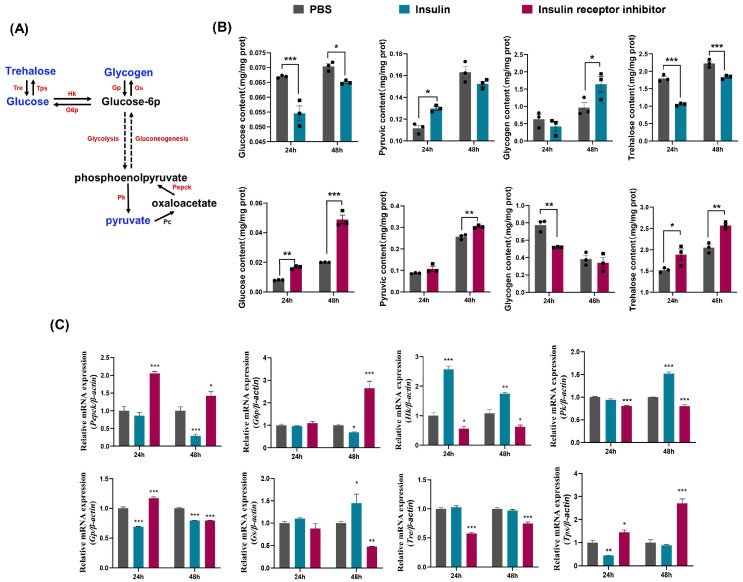
Effects of insulin and insulin receptor inhibitor on carbohydrate metabolism and gene expression in *D. suzukii*. (**A**) Schematic of carbohydrate metabolism pathways including trehalose, glucose, glycogen and pyruvate in *D. suzukii*. Trehalose is converted to glucose by trehalase (Tre), while trehalose-phosphate synthase (Tps) converts glucose to trehalose. Glucose can be phosphorylated to glucose-6-phosphate (G6p) by hexokinase (Hk) or stored as glycogen via glycogen synthase (Gs) and glycogen phosphorylase (Gp). Glucose-6-phosphate can be further metabolized through glycolysis to phosphoenolpyruvate (PEP) and pyruvate by pyruvate kinase (Pk) or enter gluconeogenesis to form glucose. Pyruvate can also be converted to oxaloacetate by pyruvate carboxylase (Pc) and further to PEP by phosphoenolpyruvate carboxykinase (Pepck). (**B**) ELSA analysis of glucose, pyruvic acid, glycogen, and trehalose levels under PBS (gray, square), insulin (blue, circle), and insulin receptor inhibitor (magenta, circle) at 24 h and 48 h. (**C**) qRT-PCR showing the mRNA expression profiles of key metabolic enzymes (*Pepck*, *G6p*, *HK*, *Pk*, *Gp*, *Gs*, *Tre* and *Tps*) at 24 h and 48 h. Data represent means ± SE, with statistical significance indicated (* *p* < 0.05, ** *p* < 0.01, *** *p* < 0.001).

To further investigate the regulatory mechanism of insulin on sugar metabolism in *D. suzukii*, we treated adult flies within 6 h of eclosion with insulin and insulin receptor inhibitor. We measured key metabolites and the transcription levels of critical genes involved in glucose-centered sugar metabolism. Glucose is a critical energy source during insect growth and development, where it is transformed into glucose-6 phosphate to enter various metabolic pathways. Glucose is stored as glycogen and trehalose and metabolized to pyruvate via glycolysis for energy production through the TCA cycle (Figure 2A). After treatment with insulin, the glucose content decreased significantly after 24 h and 48 h, whereas the pyruvate content increased at 24 h (Figure 2B). By contrast, after treatment with insulin receptor inhibitor, the glucose content increased significantly after 24 h and 48 h, whereas the pyruvate content decreased at 48 h (Figure 2B). We also measured the glycogen content, an energy storage molecule that is synthesized from glucose. The concentration of glycogen increased 48 h after insulin treatment, whereas it increased 24 h after treatment with insulin receptor inhibitor (Figure 2B). Trehalose is the primary circulating sugar in insect hemolymph, and the trehalose decreased significantly following insulin treatment but increased significantly after treatment with insulin receptor inhibitor (Figure 2B). Insulin administration promoted the conversion of glucose into pyruvate and glycogen and inhibited trehalose production. Treatment with insulin receptor inhibitor promoted the reverse to enhance gluconeogenesis and trehalose synthesis while reducing glycogen formation, thereby highlighting the pivotal role of insulin in regulating critical metabolic pathways for glucose utilization and energy storage.

To determine whether the levels of genes related to glycometabolism were correlated with fluctuations in the carbohydrate contents after treatment with insulin and insulin receptor inhibitor, we conducted qRT-PCR to assess the expression levels of genes encoding key glycolytic enzymes comprising *Hk* and *Pk*, as well as key gluconeogenic enzymes comprising *G6p* and *Pepck*. The results indicated that insulin treatment significantly increased the transcription levels of the glycolytic enzymes *Hk* and *Pk* (Figure 2C) but significantly decreased the levels of the gluconeogenic enzymes *Pepck* and *G6p* (Figure 2C). By contrast, treatment with insulin receptor inhibitor decreased the transcription levels of *Hk* and *Pk* but increased the levels of *Pepck* and *G6p* (Figure 2C). These observations demonstrated that exogenous insulin application promoted glycolysis and inhibited gluconeogenesis, which corresponded with reduced glucose levels and increased pyruvate levels. By contrast, treatment with insulin receptor inhibitor promoted gluconeogenesis and inhibited glycolysis, thereby resulting in higher glucose levels and lower pyruvate levels.

We further investigated the expression levels of key enzymes involved in glycogen and trehalose metabolism, glycogen synthase (Gs) and trehalose-6-phosphate synthase (Tps) for synthesis, and glycogen phosphorylase (Gp) and trehalase (Tre) for degradation. After insulin injection, the expression of *Gp* was significantly inhibited, whereas *Gs* was significantly activated (Figure 2C). The expression of *Tps* was also inhibited, but no significant change was observed for *Tre* (Figure 2C). These findings indicated that insulin promoted the synthesis and accumulation of glycogen but inhibited trehalose synthesis. Conversely, when insulin receptor inhibitor was applied, the *Gp* transcription levels increased significantly after 24 h but decreased after 48 h (Figure 2C). The expression of *Gs* was significantly inhibited. The *Tps* transcription levels increased significantly, whereas *Tre* was significantly inhibited (Figure 2C). These results suggested that insulin receptor inhibitor suppressed glycogen synthesis but promoted the synthesis and accumulation of trehalose. Overall, our data demonstrate the roles of insulin in modulating carbohydrate metabolism, enhancing glycogen storage, and reducing trehalose production, whereas these effects were reversed by insulin receptor inhibitor.

### 2.3. Insulin Affects FoxO Phosphorylation and Regulates FoxO Nuclear Localization via the Insulin Signaling Pathway

**Figure 3:**
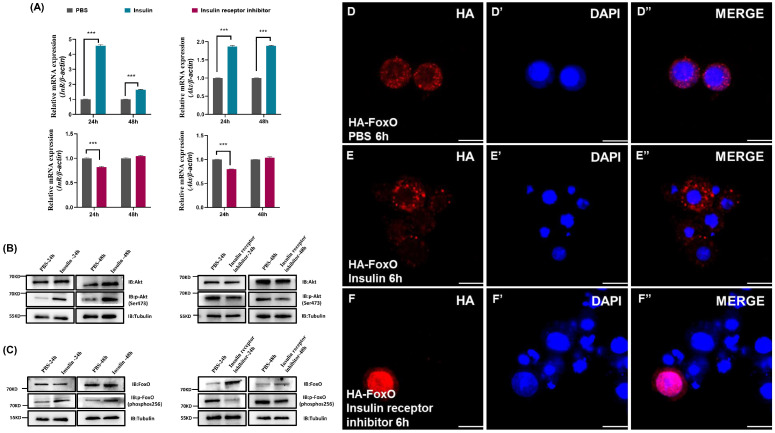
Insulin affects FoxO phosphorylation and regulates FoxO nuclear localization by the insulin signaling pathway. (**A**) qRT-PCR showing the mRNA expression profiles of *InR* and *Akt* in *D. suzukii*. Western blot analysis of total and phosphorylated levels of Akt (**B**) and FoxO (**C**) following 24 h and 48 h of insulin and insulin receptor inhibitor treatment in *D. suzukii*, with β-Actin as protein control after 8% SDS-PAGE. Immunofluorescence images showing FoxO-HA (HA, red) and nuclei (DAPI, blue) in cells treated with PBS (**D**–**D″**), insulin (**E**–**E″**), or insulin receptor inhibitor (**F**–**F″**) for 6 h. Scale bars: 20 μm. *** *p* < 0.001.

To explore the impacts of insulin and insulin receptor inhibitor on the insulin signaling pathway in *D. suzukii*, we measured the gene expression levels of *InR* and *Akt*, as well as the phosphorylation levels of Akt. Insulin treatment significantly increased the transcription of *InR* and *Akt* at 24 and 48 h post-administration (Figure 3A), as well as increasing the phosphorylation of Akt, which indicated the activation of insulin signaling (Figure 3B). By contrast, treatment with insulin receptor inhibitor markedly decreased the transcript levels of *InR* and *Akt* after 24 h (Figure 3A) and reduced Akt phosphorylation (Figure 3B), which indicated the decreased activation of insulin signaling. These changes demonstrate the efficacy of the inhibitor at modulating the activity of the insulin pathway.

The transcription factor FoxO has a critical role in IIS downstream signaling [62]. Western blotting was conducted to examine the levels of FoxO protein and its phosphorylated form after the administration of exogenous insulin and insulin receptor inhibitor. The results showed that compared with the PBS control group, insulin administration significantly increased the FoxO phosphorylation levels. By contrast, the application of insulin receptor inhibitor significantly reduced the phosphorylation of FoxO (Figure 3C). Immunocytochemistry experiments were performed to investigate the subcellular localization of FoxO in S2 cells. In the PBS control group, FoxO was distributed throughout the entire cell, including the cytoplasm and nucleus (Figure 3D). Compared with the PBS control group, insulin stimulation for 6 h increased the translocation of FoxO to the cytoplasm and showed a punctate distribution in the cytoplasm (Figure 3E), whereas treatment with insulin receptor inhibitor for 6 h led to significant relocalization of FoxO to the nucleus (Figure 3F). These findings indicate that the insulin signaling pathway regulates the nuclear localization and phosphorylation levels of FoxO. 

### 2.4. FoxO Regulates Glycometabolism Homeostasis

**Figure 4:**
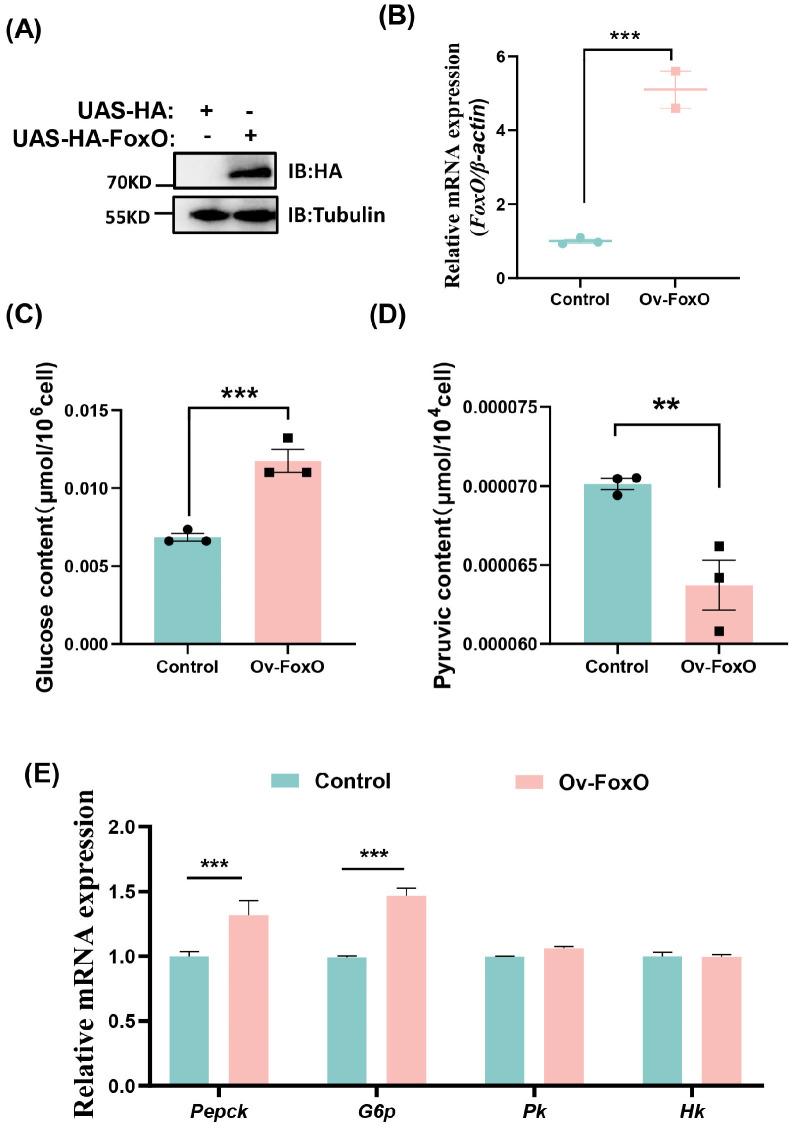
Overexpression of FoxO activates gluconeogenesis. (**A**) Western blotting validation of the FoxO overexpression efficiency in the S2 cells. (**B**) qRT-PCR showing the increased *FoxO* expression levels after overexpression of FoxO-HA in S2 cells. (**C**,**D**) The levels of glucose and pyruvate in the S2 cells. (**E**) The expression profiles of *Pepck*, *G6p*, *Pk* and *Hk* in the S2 cells. Data represent means ± SE, with statistical significance indicated (** *p* < 0.01, *** *p* < 0.001).

**Figure 5:**
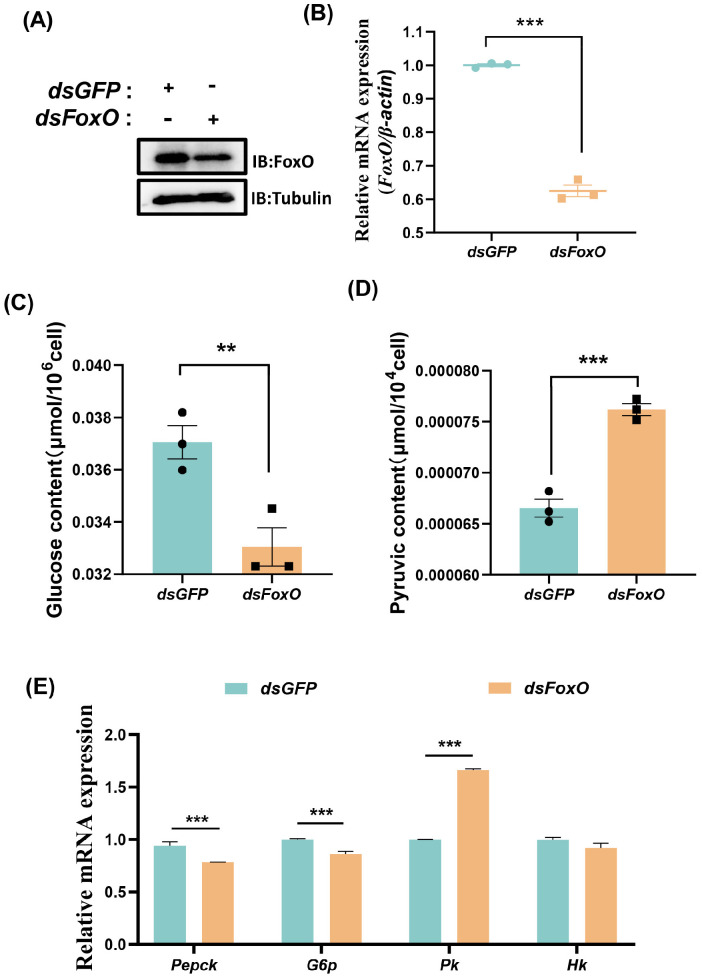
Knockdown of *FoxO* activates glycolysis. (**A**) Western blotting validation of the interference efficiency of FoxO in the S2 cells. (**B**) qRT−PCR showing the decreased *FoxO* expression levels after treatment with *dsFoxO* in S2 cells. (**C**,**D**) The levels of glucose and pyruvate in the S2 cells. (**E**) The expression profiles of *Pepck*, *G6p*, *Pk* and *Hk* in the S2 cells. Data represent means ± SE, with statistical significance indicated (** *p* < 0.01, *** *p* < 0.001).

To explore the function of FoxO in glycometabolism, we overexpressed *FoxO* by transfecting S2 cells with pUAS-FoxO-HA. Western blotting and qRT-PCR showed that the FoxO protein expression and mRNA levels were successfully activated in S2 cells (Figure 4A,B). At 48 h after transfection with pUAS-FoxO-HA, the glucose content significantly increased compared with the control group (Figure 4C), whereas the pyruvate content decreased significantly (Figure 4D). The transcriptional levels of the key enzymes associated with gluconeogenesis comprising *Pepck* and *G6p* were significantly higher, whereas the mRNA levels of the key enzymes associated with glycolysis comprising *Hk* and *Pk* were not significantly different (Figure 4E). To determine whether FoxO is responsible for gluconeogenesis activation, we performed RNAi of FoxO and analyzed the glucose and pyruvate contents. After the knockdown of *FoxO* (Figure 5A,B), the pyruvate levels increased significantly (Figure 5D) whereas the glucose contents did not change significantly (Figure 5C). Moreover, the mRNA levels of *Pepck* and *G6p* associated with gluconeogenesis decreased, whereas the mRNA levels of *Pk* associated with glycolysis increased (Figure 5E). These results suggest that FoxO may regulate glycometabolism homeostasis by upregulating the expression levels of related genes.

### 2.5. FoxO Promotes Pepck Expression to Activate Gluconeogenesis

**Figure 6:**
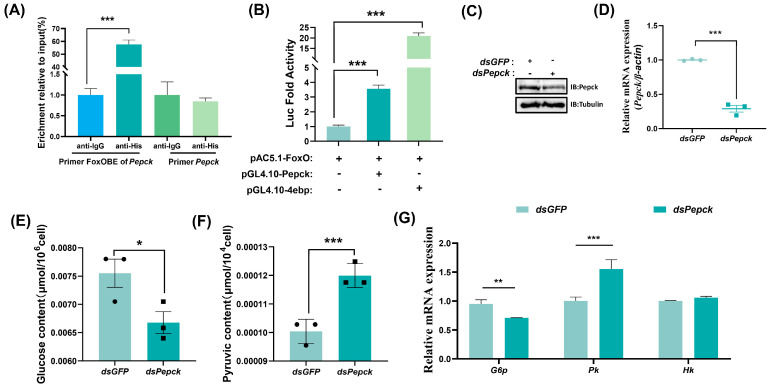
FoxO promotes *Pepck* expression to activate gluconeogenesis. (**A**) ChIP analysis of FoxO binding to the FoxOBE in the *Pepck* promoter region. Input: nonimmunoprecipitated chromatin. IgG, nonspecific rabbit IgG. Primer FoxOBE of *Pepck*: primers targeted at the *Pepck* FoxOBE-containing sequence. Primer *Pepck*: primers targeted at *Pepck* ORF. (**B**) Luciferase reporter assay after cotransfection of expression vectors pAc5.1-FoxO and reporter constructs indicates FoxO promoted *Pepck* expression and 4ebp was used as a positive control. (**C**) Western blotting validation of the interference efficiency of *Pepck* in the S2 cells. (**D**) qRT−PCR showing decreased *Pepck* expression levels after treatment with *dsPepck* in S2 cells. (**E**,**F**) The levels of glucose and pyruvate in the S2 cells. (**G**) The expression profiles of *G6p*, *Pk* and *Hk*. Data represent means ± SE, with statistical significance indicated (* *p* < 0.05, ** *p* < 0.01, *** *p* < 0.001).

Phosphoenolpyruvate carboxykinase (Pepck) serves as a key enzyme in gluconeogenesis, where its activity is primarily regulated through transcriptional control. A FoxO-binding element (FoxOBE) comprising 5′-TTGTTAAC-3′ (−1908 to −1900 bp, relative to ATG) was predicted in the promoter region of *Pepck*, and the ChIP results showed that FoxO-His bound more FoxOBE than lgG (Figure 6A). To verify the results obtained by ChIP analysis, we conducted a luciferase reporter assay, and the results showed that FoxO could bind to the *Pepck* promoter to increase the expression of *Pepck* (Figure 6B). *Pepck* was knocked down by dsRNA to further confirm its role in the regulation of glycometabolism homeostasis (Figure 6C,D), where the glucose levels decreased (Figure 6E) and pyruvate appeared to accumulate significantly (Figure 6F). After knocking down *Pepck*, the *G6p* mRNA levels decreased with gluconeogenesis. By contrast, the mRNA levels increased for *Pk* involved with glycolysis, and no significant changes occurred in the expression levels of *Hk* involved with glycolysis (Figure 6G). These results indicated that Pepck plays a key role in maintaining glucose homeostasis, with its expression being modulated by FoxO binding, thereby influencing gluconeogenesis and glycolysis pathways.

## 3. Discussion

Carbohydrate metabolism in insects is intricately regulated by hormonal signals, transcription factors, secondary messengers, and post-transcriptional modifications [63,64,65]. One of the central hormones in this regulatory network is insulin [66], which plays a crucial role in maintaining glucose homeostasis by modulating pathways such as glycolysis and gluconeogenesis [67,68,69]. Many studies have elucidated the role of insulin in mammalian metabolism, but comparatively few have investigated its regulatory mechanisms in *D. suzukii*. A recent study found differences in the egg-laying preferences of *D. suzukii* and *D. melanogaster* according to high sucrose concentrations [70]. Our findings suggest that *D. suzukii* exhibits higher adaptability to high-sugar environments, which further suggests that this species may possess flexible metabolic regulatory mechanisms to cope with varying nutritional conditions. This adaptability is likely closely related to the regulation of its insulin signaling pathway, the FoxO transcription factor, and glucose metabolism-related genes. These insights are crucial for advancing our understanding of insect survival strategies and metabolic regulation in fluctuating environments [71]. Additionally, high-sugar environments stimulate insulin secretion, suggesting that *D. suzukii* adaptation may be closely related to the regulation of its insulin signaling pathway, FoxO transcription factors and glucose metabolism-related genes. Despite the established importance of insulin in metabolic homeostasis, the precise molecular mechanisms associated with its regulatory effects on sugar metabolism in *D. suzukii* remain inadequately understood [72,73]. By elucidating these mechanisms, our findings provide insights into the adaptive strategies employed by *D. suzukii* in response to sugar-rich environments as well as helping to understand the molecular basis of metabolic diseases.

During insect development, insulin plays significant roles in the regulation of metabolism, growth, and reproduction, and it affects the lifespan of insects [74,75,76]. Glycometabolism is regulated by various hormones in different species [77,78]. For example, in the Chinese Oak Silkworm *Antheraea pernyi*, insulin decreases the hemolymph trehalose levels by promoting its uptake into tissues and conversion into glucose, which fuels glycolysis and other metabolic processes [79]. Similarly, in the mosquito *Aedes aegypti*, insulin regulates glycogen and lipid metabolism, ensuring that the energy supply is sufficient for egg production and other vital functions [80]. In the beet armyworm *Spodoptera exigua*, JH was shown to interact with ILPs and AKH to coordinate the utilization and storage of carbohydrates during different developmental stages and physiological states [81]. In humans, blood glucose levels are maintained by increasing the synthesis and storage of glycogen and utilizing glucose through glycolysis in tissue cells [82]. Based on these studies, we investigated the regulatory mechanisms of insulin on sugar metabolism in *D. suzukii* by administering insulin and insulin receptor inhibitor. ELISA and qPCR analyses demonstrated that the sugar levels and expression levels of genes encoding glycometabolism enzymes were regulated by exogenous insulin signaling in order to maintain glucose metabolic homeostasis in *D. suzukii*. Exogenous insulin activated glycolysis and promoted glucose catabolism into pyruvate, whereas glycogenolysis and trehalose synthesis were inhibited. By contrast, insulin receptor inhibitor activated gluconeogenesis to promote the synthesis of glucose from pyruvate and increase glycogen utilization and trehalose storage. These findings indicate that the dynamic balance between glycolysis and gluconeogenesis is regulated by insulin signaling.

We demonstrated that insulin functions as a major regulatory switch to govern metabolic homeostasis in *D. suzukii*. The insulin signaling pathway is an evolutionarily conserved phosphorylation cascade in both vertebrates and invertebrates [83]. In insects, insulin-like peptides (ILPs) specifically activate the insulin receptor (InR), triggering the phosphorylation of insulin receptor substrates, a process mediated by InR [84], and phosphorylation of the insulin receptor substrate is mediated by the insulin receptor [85]. This activation initiates downstream signaling cascades, primarily the phosphoinositide 3-kinase (PI3K)-serine/threonine kinase (Akt) pathway or the mitogen-activated protein kinase (MAPK) pathway [86,87,88]. We demonstrated that exogenous insulin activated the InR/Akt pathway, whereas insulin receptor inhibitor suppressed the InR/Akt pathway. Research on FoxO, a key downstream target of the insulin signaling pathway, is relatively scarce in *D. suzukii*. In mammals, insulin binds to its receptor, leading to phosphorylation and altered subcellular localization of FoxO, which in turn modulates its functional activity [89]. At high insulin levels, Akt phosphorylates FoxO, retaining it in the cytoplasm and preventing its transcriptional activity [90]. Conversely, when insulin signaling is inhibited, PI3K/Akt is deactivated, preventing Akt’s recruitment to the plasma membrane [91,92]. As a result, FoxO is not phosphorylated, allowing its translocation into the nucleus to initiate gene transcription [93]. Our study aligns with previous findings and further substantiates the regulatory role of the insulin signaling pathway in modulating FoxO activity in *D. suzukii*. This underscores the conserved nature of the insulin-mediated regulation of FoxO across different species, reinforcing its evolutionary significance. Interestingly, after 6 h of insulin treatment, FoxO was predominantly localized in the cytoplasm and exhibited a punctate distribution. FoxO serves as a mediator of stress adaptation to maintain cellular and organismal homeostasis in vivo by regulating the stress response pathway [94]. RNA granules are highly dynamic membrane-less organelles mainly comprised of stress granules, P-bodies, P-granules and neuronal granules, which perform different functions in the cell [95]. Research has shown that reducing insulin signaling pathway activity not only extends the lifespan of *Caenorhabditis elegans* but also increases the formation of RNA stress granules and P-bodies [96]. Therefore, we hypothesize that under various stress conditions, FoxO may localize to RNA granules to help regulate the fate of specific mRNAs, either by repressing translation or promoting mRNA degradation, thereby optimizing protein synthesis in response to stress and maintaining cellular homeostasis. Furthermore, FoxO transcription factors play significant roles in mediating the effects of insulin on gene expression and metabolism [97]. Genetic mutations in FoxO genes or abnormal expression levels of FoxO proteins are associated with metabolic disease, cancer, or altered lifespans in humans and animals [98]. Our experimental analyses based on the overexpression and knockdown of FoxO demonstrated its pivotal role in regulating the dynamic balance between gluconeogenesis and glycolysis to maintain glucose metabolic homeostasis in insects. In particular, the overexpression of FoxO led to the upregulation of gluconeogenesis genes and increased glucose production. By contrast, the knockdown of FoxO enhanced the expression of glycolytic genes, promoting the utilization of glucose. These findings highlight the dual regulatory roles of FoxO, where its activation shifts the metabolic balance toward gluconeogenesis and ensuring the availability of glucose during periods of nutrient scarcity or high energy demand. In addition, reducing the activity of FoxO favors glycolysis, supporting efficient glucose consumption and energy production under conditions of nutrient abundance. This dynamic regulation by FoxO is crucial for maintaining metabolic flexibility and homeostasis throughout the life cycle of insects, particularly during developmental transitions such as metamorphosis. The ability of FoxO to modulate the expression levels of key metabolic genes highlights its role as a central integrator of insulin signaling and metabolic responses. By finely tuning the balance between gluconeogenesis and glycolysis, FoxO ensures that energy production is aligned with physiological demands to sustain overall metabolic stability. In addition, studies in *Bombyx mori* have shown that the overexpression of FoxO promotes glucose synthesis and enhances fat breakdown [99], whereas in *Drosophila melanogaster*, the regulation of glycogen metabolism by FoxO highlights its broad role in carbohydrate metabolism [100].

Previous studies have demonstrated that FoxO could regulate the transcription of *IGFBP-1*, *Pepck*, and other genes through a conserved insulin response element (IRE) containing a common 5′T (G/A) TTT3′ (or 5′AAA (T/C) A3′) motif [101,102]. In mammals, FoxO1 directly regulates the transcription of *Pepck* and *G6Pase*, key enzymes in gluconeogenesis [103]. In *Caenorhabditis elegans*, DAF-16 has been shown to participate in the gluconeogenesis pathway by upregulating *Pepck* expression, promoting both longevity and metabolic health [104]. In *Bombyx mori*, FoxO enhances disease resistance by upregulating *PCK2* [105]. We found that *Pepck* transcript levels were significantly increased when FoxO was overexpressed and significantly decreased when FoxO was disrupted, which is consistent with previous reports. In *D. melanogaster*, *Pepck* is predicted to be a direct target of FoxO [106]. However, the regulation of *Pepck* by FoxO remains controversial, as studies show that insulin inhibits *Pepck* transcription independently of the IRS, suggesting that FoxO’s role in this process may not rely on IRS binding [107,108]. Our findings provide direct experimental evidence confirming this relationship through Chip-PCR and luciferase reporter experiments in *D. suzukii*. This study not only confirms the regulatory role of FoxO on *Pepck* but also highlights the evolutionary conservation of this mechanism across species. This research deepens our understanding of the FoxO-Pepck axis, which is crucial for gluconeogenesis, metabolic health, and potentially pest control applications in agricultural settings. Pepck catalyzes the rate-controlling step in gluconeogenesis, and it is thus a central player in glucose homeostasis [109]. Knockdown of *Pepck* led to an increase in the glycolytic activity, thereby suggesting that Pepck acts as a regulatory node between gluconeogenesis and glycolysis. Thus, by upregulating *Pepck*, FoxO may enhance gluconeogenesis, which is balanced by its role in modulating glycolytic pathways to maintain glucose homeostasis. The direct binding of FoxO to the *Pepck* promoter and the subsequent regulation of *Pepck* expression highlight a specific molecular pathway that allows FoxO to influence glucose metabolism. Our findings suggest that FoxO is a central mediator in metabolic homeostasis and that the FoxO–Pepck axis is crucial for balancing glucose production and utilization, thereby ensuring that metabolic demands are met under varying physiological conditions.

## 4. Materials and Methods

### 4.1. Experimental Insects and S2 Cells

*D. suzukii* individuals were sourced from a cherry orchard in Jinan, Shandong Province, China. The flies were kept in a controlled environment at 25 °C under a 12 h light/dark cycle and provided with a cornmeal–agar diet. The artificial diet was prepared by mixing 60 g of corn flour, 40 g of sucrose, 50 g of yeast extract, 7 g of agar, and water to make a total of 1 L. For the low-sugar diet, sucrose was reduced to 20 g, while for the high-sugar diet, it was increased to 60 g. S2 cells were cultured in cell culture flasks, utilizing both suspension and adherent growth, and maintained in SFX Drosophila medium (Hyclone, Logan, UT, USA) supplemented with 1% penicillin/streptomycin, with incubation at 25 °C.

### 4.2. Detection of Life History Traits

To evaluate the survival rates of *D. suzukii* under varying dietary conditions, we organized three experimental groups: low sugar, control, and high sugar. Each group comprised 20 adult flies, with 3 biological replicates per dietary condition, totaling 60 flies for each diet. Dead flies were counted every 6 days, and survival rates for each group were computed. Kaplan–Meier plots were used to analyze the survival curves.

For assessing the oviposition rate, newly emerged adult flies were immediately paired into groups of five male–female pairs. Each dietary treatment included three replicates, amounting to 15 pairs per condition. The total number of eggs deposited by each group was recorded over a 10-day period.

### 4.3. Hormone Stimulation

Insulin (Solarbio, Beijing, China) was diluted to 0.1 mg/mL and HNMPA-(AM)3 (Abcam, Cambridge, UK) was diluted to 2.5 μM with phosphate-buffered saline (PBS) for storage at −20 °C. For the experimental procedure, adult flies that had eclosed 6 h prior were injected with either insulin or HNMPA-(AM)3 and then cultured for 24 h or 48 h. Control groups received an equivalent volume of PBS and underwent the same incubation periods. Additionally, S2 cells were transfected with pUAS-FoxO-HA plasmids and, after 48 h, treated with insulin, HNMPA-(AM)3, or PBS as a control. The samples prepared from these treatments were utilized in subsequent experiments, each of which was conducted in triplicate.

### 4.4. Insulin Determination

Each treatment group consisted of six flies per replicate, and each substance was tested in three separate biological replicates. To determine insulin concentrations in *D. suzukii* under different sugar conditions, we used the Insulin assay kit (mlbio, Shanghai, China) following the manufacturer’s instructions. Absorbance was measured at 450 nm.

### 4.5. Glucose Determination

To measure glucose levels, we analyzed both *D. suzukii* and S2 cell samples. Each *D. suzukii* sample was composed of six flies per group, while S2 cells samples consisted of a 3 mL cell suspension that was centrifuged at 2000× *g* for 3 min. For each type of sample, three independent biological replicates were performed. The *D. suzukii* samples were homogenized with 500 µL of PBS, whereas S2 cells samples were homogenized in 200 µL of PBS. Glucose levels were measured using a glucose assay kit (Sigma, St. Louis, MO, USA), and absorbance was recorded at 540 nm. 

### 4.6. Glycogen Determination

To quantify glycogen, samples from various *D. suzukii* treatments were subjected to an extraction using the alkaline buffer from the Glycogen Assay Kit (Solarbio, China). This process adhered to the same protocol employed for insulin measurement. The samples were heated in a water bath at 100 °C for 20 min, after which they were allowed to cool. Following cooling, the remaining reagents were added in sequence according to the instructions provided by the kit. Absorbance readings were taken at 620 nm using a spectrophotometer.

### 4.7. Trehalose Determination

The method for sample collection adhered to the previously outlined procedure. Each sample was mixed with 500 µL of extraction buffer and left at room temperature for 45 min. Trehalose concentrations were determined using a detection kit from Solarbio (China). After allowing the samples to cool, absorbance was measured at 620 nm.

### 4.8. Pyruvate Determination

The procedure for pyruvate measurement was analogous to that employed for glucose sample analysis. *D. suzukii* samples were processed by homogenizing them in 600 µL of PBS, while S2 cells samples were homogenized in 300 µL of PBS. Following the instructions provided by the pyruvate assay kit (Solarbio, China), the absorbance of the samples was measured immediately at 520 nm.

### 4.9. qRT-PCR

The samples were homogenized in TRIzol reagent, followed by the addition of chloroform to separate nucleic acids, and isopropanol to precipitate RNA. The resulting RNA pellet was rinsed with 75% ethanol and resuspended in RNase-free water. Once the RNA concentration was measured, any remaining DNA was removed using a cDNA synthesis kit from TaKaRa. A total of 1 µg of RNA was then reverse-transcribed into single-strand cDNA. Quantitative RT-PCR (Vazyme, Nanjing, China) was conducted in a 20 µL reaction mixture, with β-actin (Actb) used as the internal control gene.

### 4.10. Western Blotting

*D. suzukii* samples were homogenized in RIPA buffer supplemented with protease and phosphatase inhibitors to safeguard protein integrity. The homogenates were then subjected to centrifugation at 12,000 rpm for 10–15 min at 4 °C, and the supernatants were carefully collected. To denature the proteins, an equal volume of 1× SDS loading buffer was added to the samples, which were then heated at 100 °C for 5 min. The denatured proteins were separated by loading the samples onto a polyacrylamide gel and performing electrophoresis according to their molecular weights. Following separation, the proteins were transferred from the gel onto a PVDF or nitrocellulose membrane. The membrane was blocked with 5% non-fat dry milk and then incubated with the primary and secondary antibodies for 2 h each at room temperature. The membrane was washed with TBST between antibody incubations. Protein bands were detected using ECL luminescence reagent and visualized through a fluorescence detection system.

### 4.11. RNAi in the S2 Cells Line

Double-stranded RNA (dsRNA) was generated utilizing the T7 RiboMAX™ Express RNAi System (Promega, Madison, WI, USA), in accordance with the manufacturer’s guidelines. For transient transfections, lip3000 (Thermo, Waltham, MA, USA) was used following the prescribed protocol. The dsRNA and Lip3000 were applied at final concentrations of 2 µg/mL and 4 µg/mL, respectively. The control group was treated with an equivalent quantity of dsGFP. The analysis included evaluating RNA interference effectiveness, examining key gluconeogenesis enzymes, and quantifying glucose and pyruvate levels.

### 4.12. Immunocytochemistry

After transfecting cells with pUAS-FoxO-HA for 48 h, the cells were exposed to insulin and HNMPA-(AM)3 for 6 h. Following this treatment, S2 cells were collected by low-speed centrifugation. The cells were fixed with 4% formaldehyde and then incubated with the primary and secondary antibodies for 2 h each at room temperature. After washing the cells with PBST, the nuclei were stained with DAPI. Finally, the cells were mounted using an anti-fade reagent and imaged using a Zeiss confocal microscope.

### 4.13. ChIP-PCR

The pUAS-FoxO-His plasmid was transfected into S2 cells for 48 h. The cells were cross-linked by incubating with 4% formaldehyde at room temperature for 10 min, followed by glycine to quench the reaction, and then washed with PBS. The S2 cells were lysed using RIPA buffer, and chromatin was fragmented via sonication or enzymatic digestion. The resulting chromatin fragments were incubated with specific antibodies overnight at 4 °C. Immunoprecipitation was performed using Protein A/G agarose beads. The samples were sequentially washed with buffers to remove non-specific interactions. The DNA was purified by phenol/chloroform extraction and analyzed by qRT-PCR using FoxOBE-F/FoxOBE-R primers (Table S1).

### 4.14. Luciferase Reporter Assay

The *Pepck* promoter region was amplified via PCR and subsequently cloned into the pGL4.10 reporter vector. A positive control plasmid for 4ebp was also constructed. The FoxO coding sequence was inserted into the His-tagged expression plasmid pAc5.1. Both the reporter plasmid and the positive control were co-transfected into S2 cells with Lip2000 transfection reagent. After a 48 h incubation period, luciferase activity was assessed using a dual-luciferase assay kit (Vazyme, China) following the manufacturer’s guidelines.

### 4.15. Date Analysis

Each experiment was repeated independently three times, and the values were expressed as the mean ± SE based on three independent experiments. All data analyses were performed using GraphPad Prism 9. The data were analyzed by Student’s *t*-test for data difference, and *p*-values were considered to indicate significant differences at: * *p* ≤ 0.05; ** *p* ≤ 0.01; *** *p* ≤ 0.001.

## 5. Conclusions

**Figure 7:**
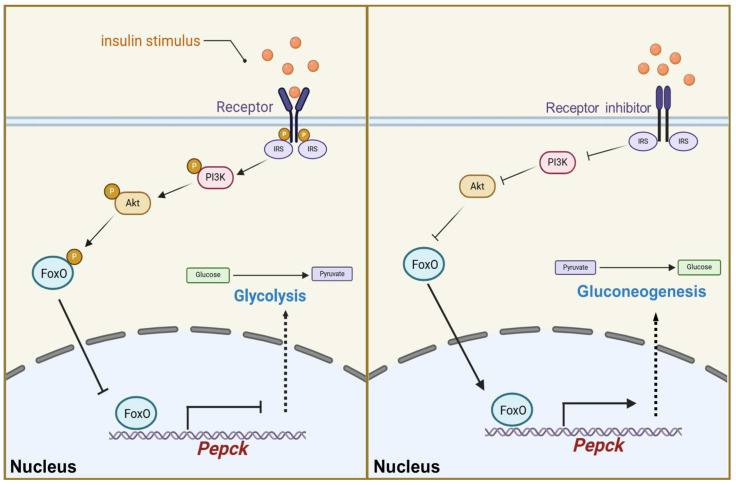
A diagram illustrating how the insulin signaling pathway regulates glucose homeostasis via FoxO-Pepck axis. When the insulin signaling pathway is activated, phosphorylated Akt (p-Akt) reduces FoxO activity by phosphorylating FoxO. This action decreases the transcription of Pepck, resulting in the activation of glycolysis and a decrease in glucose levels. Conversely, when the insulin signaling pathway is inhibited, Akt activity is suppressed, allowing FoxO to translocate into the nucleus. This facilitates the transcription of Pepck, leading to the activation of gluconeogenesis and an increase in glucose levels.

Our results demonstrate that insulin signaling plays a crucial role in regulating glucose metabolism homeostasis in *D. suzukii*. Exogenous insulin activated the insulin signaling pathway, promoting FoxO phosphorylation and reducing its activity as a transcription factor, which in turn lowered *Pepck* transcription levels, thereby activating glycolysis and inhibiting gluconeogenesis. By contrast, treatment with insulin receptor inhibitor suppressed the insulin signaling pathway, increased the nuclear localization of FoxO, and elevated the *Pepck* transcription levels, thereby activating gluconeogenesis and inhibiting glycolysis (Figure 7). These findings provide insights into the molecular mechanisms by which insulin signaling modulates glucose metabolism and highlight the significant role of the FoxO–Pepck axis in maintaining glucose homeostasis. These insights into the regulatory pathway suggest potential targets for metabolic interventions aimed at controlling the pest species *D. suzukii*.

## Data Availability

The data that support the findings of this study are presented within the manuscript and Supplemental Materials, and additional information is available from the corresponding author upon reasonable request.

## Supplementary Materials

The following supporting information can be downloaded at https://www.mdpi.com/article/10.3390/ijms251910441/s1.
