# Supplementary material for: Insulin Signaling Pathway Mediates FoxO–Pepck Axis Regulation of Glucose Homeostasis in Drosophila suzukii
Source: Int J Mol Sci. 2024 Sep 27;25(19):10441. doi: 10.3390/ijms251910441 (PMC11482478; doi:10.3390/ijms251910441)
Supplement: Supplementary file 1 [file ijms-25-10441-s001.zip › ijms-3098640-supplementary.pdf]

Table S1. PCR primer sequences and GenBank accession numbers of genes used in the experiments.

Primer name	Sequence (5'-3')	GenBank accession number
**qRT-PCR**		
Tre-D. suzukii-RTF	cgatcagttcggagtcggag	XP_016928902.1
Tre-D. suzukii-RTR	actatggtcacagctgccac	
Tps-D. suzukii-RTF	tgtgtccggtggtgatcaag	XP_016943134.1
Tps-D. suzukii-RTR	gatgttcacggacaccacct	
Gp-D. suzukii-RTF	cacctgcattacaccctggt	XP_016945120.1
Gp-D. suzukii-RTR	gagcggcccatgtagtactc	
Gs-D. suzukii-RTF	cagacaggccagggttaagg	XP_016935687.2
Gs-D. suzukii-RTR	ttcggtttcctcatccacgg	
G6p-D. suzukii-RTF	gactgtggctctgcagagtt	NP_001097063.1
G6p-D. suzukii-RTR	ggatacatgacgcagcggta	
Pepck-D. suzukii-RTF	aagggcaaggtgatcatgca	XP_016941208.1
Pepck-D. suzukii-RTR	gagttctcgccgtatccagg	
Hk-D. suzukii-RTF	gcaaccctttgtgctgagtg	NP_524674.1
Hk-D. suzukii-RTR	ggccaagtacttgcccatct	
Pk-D. suzukii-RTF	tactggtctgatcggaggca	XP_036672950.1
Pk-D. suzukii-RTR	gagatcagaccgtcgtcgac	
InR-D. suzukii-RTF	gatctggcagctcgcaattg	XP_036672528.1
InR-D. suzukii-RTR	cacatcactggcactggagt	
Akt-D. suzukii RTF	tgaaaaagataccgccgcct	XP_036673499.1
Akt-D. suzukii RTR	tccttggtagctgaactgcg	
β-actin-D. suzukii RTF	gtgctggtggagtcctacac	XP_016931735.1
β-actin-D. suzukii RTR	atatcgatgtctgccgcctg	
G6p-D. melanogaster-RTF	cgcttgagttttggaaggcc	XP_021188037.1
G6p-D. melanogaster-RTR	aggacgcgtctccggatata	
Pepck-D. melanogaster-RTF	tacgaggctcgagactggaa	NP_611341.1
Pepck-D. melanogaster-RTR	gacccagatagtcgccgaag	
Hk-D. melanogaster-RTF	acgataccactggcaccttg	ACD74807.1
Hk-D. melanogaster-RTR	acaatcacactccgcttcgt	
Pk-D. melanogaster-RTF	gcacaacctggacgagatca	NP_524448.3
Pk-D. melanogaster-RTR	actccaacatctgagtggcg	
**RNAi**		
FoxO-RNAiF	taatacgactcactataggccattagggcgcaggatctt	

FoxO-RNAiR	taatacgactcactatagggtacgtgttcagggagtccg
Pepck-RNAiF	taatacgactcactataggcccgccgagaaccttattgt
Pepck-RNAiR	taatacgactcactataggggagccagactgctttccat
GFP-RNAiF	taatacgactcactatagggcacaagttcagcgtgtccg
GFP-RNAiR	taatacgactcactataggggttcaccttgatgccgttc
**ChIP**	
FoxOBE-*Pepc*F	aaagtttacttgtttacgccact
FoxOBE-*Pepck*R	agcactacgacaataattgatatacaa
